# Prognostic model for overall survival of head and neck cancer patients in the palliative phase

**DOI:** 10.1186/s12904-023-01325-y

**Published:** 2024-02-24

**Authors:** Arta Hoesseini, Aniel Sewnaik, Boyd N. van den Besselaar, Jang Zhang, Nikki van Leeuwen, Jose A. Hardillo, Robert Jan Baatenburg de Jong, Marinella P. J. Offerman

**Affiliations:** 1 Department of Otorhinolaryngology and Head and Neck Surgery, Erasmus MC Cancer Institute, Erasmus University Medical Center, Dr. Molewaterplein 40, Rotterdam, 3015 GD The Netherlands; 2 Department of Public Health, Erasmus University Medical Center, Rotterdam, The Netherlands

**Keywords:** Prognosis, Prognostic factors, Palliative care, Head and Neck squamous cell cancer, Prediction, Survival, Prognostic model, Cox regression, Shared Decision-Making

## Abstract

**Background:**

Patients with head and neck squamous cell carcinoma (HNSCC) enter the palliative phase when cure is no longer possible or when they refuse curative treatment. The mean survival is five months, with a range of days until years. Realistic prognostic counseling enables patients to make well-considered end-of-life choices. However, physicians tend to overestimate survival. The aim of this study was to develop a prognostic model that calculates the overall survival (OS) probability of palliative HNSCC patients.

**Methods:**

Patients diagnosed with incurable HNSCC or patients who refused curative treatment for HNSCC between January 1st 2006 and June 3rd 2019 were included (n = 659). Three patients were lost to follow-up. Patients were considered to have incurable HNSCC due to tumor factors (e.g. inoperability with no other curative treatment options, distant metastasis) or patient factors (e.g. the presence of severe comorbidity and/or poor performance status).Tumor and patients factors accounted for 574 patients. An additional 82 patients refused curative treatment and were also considered palliative. The effect of 17 candidate predictors was estimated in the univariable cox proportional hazard regression model. Using backwards selection with a cut-off *P*-value < 0.10 resulted in a final multivariable prediction model. The C-statistic was calculated to determine the discriminative performance of the model. The final model was internally validated using bootstrapping techniques.

**Results:**

A total of 647 patients (98.6%) died during follow-up. Median OS time was 15.0 weeks (95% CI: 13.5;16.6). Of the 17 candidate predictors, seven were included in the final model: the reason for entering the palliative phase, the number of previous HNSCC, cT, cN, cM, weight loss in the 6 months before diagnosis, and the WHO performance status. The internally validated C-statistic was 0.66 indicating moderate discriminative ability. The model showed some optimism, with a shrinkage factor of 0.89.

**Conclusion:**

This study enabled the development and internal validation of a prognostic model that predicts the OS probability in HNSCC patients in the palliative phase. This model facilitates personalized prognostic counseling in the palliative phase. External validation and qualitative research are necessary before widespread use in patient counseling and end-of-life care.

## Background

Patients with head and neck squamous cell carcinoma (HNSCC) in general have a poor prognosis with a five-year survival rate varying between 35% and 70%, depending on the stage and location of the tumor [1]. Patients enter the palliative phase, when cure is no longer possible or when patients refuse curative treatment [2]. Patients in this phase deal with many symptoms that have a great impact on daily functioning like dyspnea, dysphagia, communication difficulties, fatigue, pain, and psychosocial complaints [3–5]. The life-span in this phase is usually short with a mean survival of five months, but with a wide individual range of days until years [2]. Palliative care improves the quality of life of patients and their families in this phase through the prevention and relief of suffering such as pain and other problems [6].

Several studies among incurable cancer patients’ have shown patients’ desire for detailed prognostic information [7–9]. Head and neck cancer patients that participated in our focus group research stressed the wish to receive quantitative prognostic information in case of cancer recurrence and a poor prognosis [10]. While patients seek prognostic information, physicians are often reluctant to discuss prognosis. One of the reasons for this is the physicians’ concern to be proven wrong. Our study among head and neck cancer patients in the palliative phase showed that in only 18% of cases survival was accurately predicted by their treating physician, while in 58% it was overestimated [11]. This tendency to overestimate is in agreement with earlier research among other cancer types [12–14]. Being able to predict survival more accurately early in the palliative phase facilitates realistic prognostic discussions and enables patients and their caregivers to make well-considered end-of-life choices. It can help in the decision whether palliative treatment is desirable. Furthermore, it enables patients and their caregivers to prepare themselves for the approaching end of life and palliative care planning can be optimized.

We have developed the prognostic model OncologIQ to predict the survival probability of HNSCC patients in the curative phase more accurately. This model, which was recently updated, calculates the 1- to 10-year overall survival (OS) probability based on several prognostic factors like age, sex, Body Mass Index (BMI), and comorbidity among HNSCC patients who are eligible for curative treatment [15–18]. As the need for prognostic information might be even higher in the palliative phase [10], we aimed to develop a prognostic model for this group. Our hypothesis is that prognostication in the palliative phase requires other predictors than in the curative phase. With this palliative model we aim to predict the individual prognosis more accurately which can lead to more realistic prognostic discussion and assist in personalized prognostic counseling in the palliative phase.

## Methods

This study was approved by the Erasmus MC Medical Ethics Review Committee (MEC number: MEC-2016-751). All methods were performed in accordance with the relevant guidelines and regulations.

### Data collection

The data used in this study were retrieved from the Rotterdam Oncological Documentation database (RONCDOC) [18]. Patients who were diagnosed with a primary HNSCC at the Erasmus MC Cancer Institute between January 1st 2006 and December 31st 2013 were included in the database. Follow-up time was last updated on the 3rd of June 2019, by consulting the Municipal Personal Records Database (MPRD). Final day of follow-up time for a patient was defined as the final date that the patient was confirmed to be alive or the date of death.

### Eligibility criteria

Patients included in the RONCDOC database diagnosed with incurable HNSCC or patients who refused curative treatment for HNSCC between January 1, 2006 and June 3, 2019 were included in this study (n = 659). Three patients were lost to follow-up, thus 656 patients remained for the analysis. Patients were considered to have incurable HNSCC due to tumor factors (e.g. inoperability with no other curative treatment options, distant metastasis) or patient factors (e.g. the presence of severe comorbidity and/or poor performance status). Tumor and patients factors accounted for 574 patients. An additional 82 patients refused curative treatment and were also considered palliative. Included tumor sites were: oral cavity, oropharynx, nasopharynx, hypopharynx, supraglottic larynx, glottic larynx and unknown primary. Patients were discussed in our multidisciplinary tumor board. In this weekly meeting, medical oncologists, head and neck surgeons, radiotherapists, radiologists, geriatricians, and physician assistants are present to discuss all patients with a head and neck cancer diagnosis, both curative and palliative. Next, the boards treatment recommendations were discussed with the patient and palliative patients were referred to our Expert Center for Palliative Care.

### Definitions of variables

All tumor- and patient specific data were scored at the date of diagnosis of the palliative tumor. cTNM classification was scored according to the 7th American Joint Committee on Cancer (AJCC) edition of the TNM classification [19]. Previous HNSCC was defined as the total number of HNSCC’s before the palliative tumor was diagnosed. Patients were considered to have incurable HNSCC due to tumor factors (e.g. inoperability with no other curative treatment options, distant metastasis) or patient factors (e.g. the presence of severe comorbidity and/or poor performance status). Also, patients who refused curative treatment and therefore entered the palliative phase were included. Tumor type was categorized as: primary, recurrent and residual tumors. Primary tumors included the first HNSCC and second primary HNSCC which developed at a different tumor site than the first tumor. Recurrent tumors included local-regional and/or metastatic HNSCC diagnosed ≥ 3 months and < 5 years after the last day of treatment of the initial tumor. Residual tumors were defined as local-regional HNSCC diagnosed < 3 months after the last day of initial treatment. The cumulative quantity of smoking was defined in pack-years (PY) in which one pack year was equal to one packet of 20 cigarettes smoked per day for one year. A patient was considered a former smoker if he or she had stopped smoking for ≥ 3 months. A patient was considered a former drinker if he or she had stopped drinking for ≥ 6 months. The number of alcohol units per week were scored according to a standardized list: one unit, or 10 g, of alcohol is equivalent to 12.5 milliliters of pure ethanol [20]. Patient’s comorbidity was scored according to the Adult Comorbidity Evaluation-27 (ACE-27) checklist, which gives a severity score (0 = none, 1 = mild, 2 = moderate, 3 = severe) for 12 different comorbidity categories, and an overall score [21]. Weight loss in kilograms (kg) was defined as weight loss in the six months before the palliative diagnosis. WHO performance status, also known as the Eastern Cooperative Oncology Group (ECOG) score, was scored according to the classification published by Oken et al. [22] Marital status was defined as being married or having a durable relationship versus being single or widowed.

### Statistical analyses

Statistical analyses were performed using IBM Statistical Package for the Social Sciences (version 25.0), and R statistical software (version 3.6.2) using the R packages *mice* and *rms*. Multiple imputation in R was used for handling missing predictor data (five iterations) that were assumed missing at random.

#### Univariable analyses

The Cox proportional hazards regression model was used to calculate the Hazard Ratios (HR) for all candidate predictors except for tumor stage. Significance was tested using the log-rank test and *P*-values of < 0.05 were considered significant. Serum hemoglobin was excluded from the analysis due to the large number of missing cases. The number of alcohol units per week was excluded because of the inability to impute properly. In total 17 predictor variables were identified for analysis.

#### Model development

The palliative model was developed using the backward selection method: 17 variables were added and excluded one by one until all variables left in the new model had a *P*-value < 0.10 (two-sided tests): sex, age, the reason for entering the palliative phase (incurable tumor / refusal), localization, the number of previous HNSCC, tumor type (primary / recurrent / residual), cTNM, ACE-27, smoking, pack years, alcohol, BMI, weight loss in the 6 months before diagnosis, WHO performance status, and marital status. The C-statistic was used to assess the models’ discriminative ability. The C-statistic takes values between 0.5 and 1.0, where 0.5 indicates that the model is not better than chance classification and 1 means perfect discrimination [23]. Generally a C-statistic below 0.6 can be considered as poor, a C-statistic over 0.6 as moderate, a C-statistic over 0.7 as good and a C-statistic over 0.8 as strong [24]. The final model was internally validated by bootstrapping resulting in an internally validated C-statistic. The shrinkage factor can be used to correct for potential overfitting which can lead to finding coincidental associations between a variable and outcome measure [24]. The shrinkage factor was calculated and the regression coefficients were multiplied by it to provide more reliable predictions for future patients. The Transparent Reporting of a multivariable prediction model for Individual Prognosis Or Diagnosis (TRIPOD) Statement was used as a guideline during the development and reporting of the model [25].

## Results

### Patient characteristics

Baseline characteristics and missing data are described in Table 1. A total of 647 patients (98.6%) died during follow-up. Median overall survival time was 15.0 weeks (95% CI: 13.5;16.6) with a range of 1–4680 days. Five patients were diagnosed with two simultaneous tumors. In total 82 patients refused curative treatment of a primary (n = 60, 73.2%), recurrent (n = 20, 24.4%) or residual (n = 2, 2.4%) tumor. A total of 192 patients (29.3%) received treatment with palliative intent: n = 150 (77.7%) received radiotherapy, n = 22 (11.5%) chemotherapy, and n = 14 (7.2%) chemoradiation. Furthermore, n = 1 (0.5%) underwent surgery, n = 3 (1.6%) underwent radiotherapy combined with hyperthermia and one patient (0.5%) refused curative treatment and underwent an alternative diet and light therapy treatment instead. Of the 192 patients who received palliative treatment, only 8 (4.2%) were patients who entered the palliative phase due to refusal of curative treatment.

**Table 1:** Patient characteristics, univariable, and multivariable analyses

Characteristic	Frequency (%)/ median (Q1-Q3)/ mean (SD)	Total no. missing (%)	Univariable HR (95% CI)	*p*	Multivariable HR (95% CI)	*p*
**Total**	656					
**Sex** (female)	179 (27.3)	0	1.0			
Male	477 (72.7)		1.04 (0.88; 1.24)	0.6402		
**Age**	65.4 (11.0)	0	0.99 (0.99; 1.00)	0.0412		
**Reason palliative phase** (incurable tumor)	574 (87.5)	0	1.0	-	1.0	
Refusal of curative treatment	82 (12.5)		0.82 (0.65; 1.03)	0.0895	1.39 (1.06; 1.82)	0.0181
**Localisation** (Oropharynx)	215 (32.8)	0	1.0	-		
Oral cavity	168 (25.6)		1.09 (0.89; 1.34)	0.4139		
Hypopharynx	114 (17.4)		0.90 (0.72; 1.13)	0.3762		
Nasopharynx	21 (3.2)		0.90 (0.58; 1.41)	0.6515		
Supraglottic larynx	68 (10.4)		1.03 (0.78; 1.35)	0.8570		
Glottic larynx	42 (6.4)		1.16 (0.83; 1.62)	0.3753		
Unknown primary	28 (4.3)		0.97 (0.66; 1.44)	0.8843		
**No. previous HNSCC** (0)	223 (34.0)	0	1.0	-	1.0	
1	305 (46.5)		1.42 (1.19; 1.70)	< 0.0001	1.76 (1.40; 2.21)	< 0.0001
2	106 (16.2)		1.31 (1.03; 1.65)	0.0249	1.73 (1.31; 2.29)	0.0001
3 / 4	22 (3.4)		1.48 (0.95; 2.29)	0.0821	2.00 (1.25; 3.20)	0.0037
**Tumor stage** (Stage I)	8 (1.2)	1 (0.2)	-			
Stage II	16 (2.4)		-			
Stage III	48 (7.3)		-			
Stage IVA	198 (30.2)		-			
Stage IVB	84 (12.8)		-			
Stage IVC	301 (46.0)		-			
**Tumor type** (primary)	242 (36.9)	0	1.0			
Recurrent	348 (53.0)		1.34 (1.13; 1.58)	0.0006		
Residual	66 (10.1)		1.50 (1.14; 1.97)	0.0039		
**cT** (0)	237 (36.2)	1 (0.2)	1.0		1.0	
1	24 (3.7)		0.58 (0.37; 0.88)	0.0111	0.77 (0.49; 1.21)	0.2614
2	51 (7.8)		0.59 (0.43; 0.80)	0.0008	0.80 (0.56; 1.15)	0.2232
3	74 (11.3)		0.81 (0.62; 1.05)	0.1092	1.10 (0.82; 1.48)	0.5144
4	269 (41.1)		0.97 (0.82; 1.16)	0.7739	1.41 (1.11; 1.78)	0.0044
**cN** (0)	332 (50.8)	2 (0.3)	1.0	-	1.0	
1	67 (10.2)		1.01 (0.78; 1.32)	0.9218	1.17 (0.89; 1.56)	0.2645
2	214 (32.7)		1.02 (0.86; 1.22)	0.7814	1.22 (0.99; 1.50)	0.0562
3	41 (6.3)		0.92 (0.66; 1.28)	0.6172	1.67 (1.17; 2.40)	0.0052
**cM** (0)	353 (54.0)	2 (0.3)	1.0	-	1.0	
1	301 (46.0)		1.23 (1.06; 1.44)	0.0080	1.51 (1.22; 1.88)	0.0001
**ACE-27** (0)	60 (9.1)	0	1.0	-		
1	80 (12.2)		0.83 (0.59; 1.17)	0.2870		
2	311 (47.4)		1.26 (0.96; 1.67)	0.0990		
3	205 (31.3)		1.48 (1.11; 1.98)	0.0080		
**Smoking** (current)	258 (44.1)	71 (10.8)	1.0	-		
former	262 (44.8)		0.95 (0.80; 1.12)	0.5326		
non-smoker	65 (11.1)		0.78 (0.59; 1.03)	0.0847		
**Pack years**	39.0 (22.0–50.0)	217 (33.1)	1.002 (0.998; 1.005)	0.2239		
**Alcohol** (current)	268 (50.6)	126 (19.2)	1.0	-		
former	163 (30.8)		1.05 (0.87; 1.26)	0.6252		
non-drinker	99 (18.7)		0.88 (0.69; 1.13)	0.3332		
**No. alcohol units** per week	14.0 (2.0–35.0)	223 (34.0)	-			
**Body Mass Index**	22.1 (4.4)	126 (19.2)	0.972 (0.948; 0.997)	0.0266		
**Weight loss in the 6 months before diagnosis** kg*	5.0 (0.0–9.0)	157 (23.9)	1.03 (1.02; 1.04)	< 0.0001	1.03 (1.01; 1.04)	< 0.0001
**WHO performance** (0)	92 (16.2)	89 (13.6)	1.0		1.0	
1	261 (46.0)		1.54 (1.21; 1.96)	0.0005	1.54 (1.19; 2.00)	0.0009
2	130 (22.9)		1.79 (1.37; 2.34)	< 0.0001	1.80 (1.37; 2.36)	< 0.0001
3 / 4	84 (14.8)		3.68 (2.65; 5.11)	< 0.0001	4.16 (2.95; 5.85)	< 0.0001
**Marital status** (alone)	265 (41.3)	14 (2.1)	1.0			
married/together	377 (58.7)		1.03 (0.88; 1.20)	0.7544		
**Serum Hemoglobin** mmol/L **	7.7 (1.3)	409 (62.3)	-			
**Internally validated C index**					0.66	

* 1 kg (kg) = 2.20 pounds

** Hemoglobin of 7.7 mmol/L = 12.4 g/dl

### Univariable analysis

Nine out of seventeen analyzed candidate predictors showed a univariable significant effect on OS (see Table 1). Patients who refused curative treatment had a better survival probability than patients with an incurable head and neck tumor (HR 0.82, 95% CI 0.65; 1.03), *P* = 0.0895). These potential curative patients were significantly older, had significant less previous HNSCC, and significant lower ACE-scores. Patients who had already been treated for one or two HNSCC had a significant worse survival probability compared to patients with no history of HNSCC. The same accounted for patients with a recurrent (HR 1.34, 95% CI: 1.13; 1.58) or residual tumor (HR 1.50, 95% CI 1.14; 1.97), M1 tumor, ACE-score of 3 and more weight loss in the 6 months before diagnosis. A higher BMI was significantly associated with a better survival probability. WHO performance status showed a significantly worse survival probability with an increasing HR per category.

### Model development

**Fig. 1:**
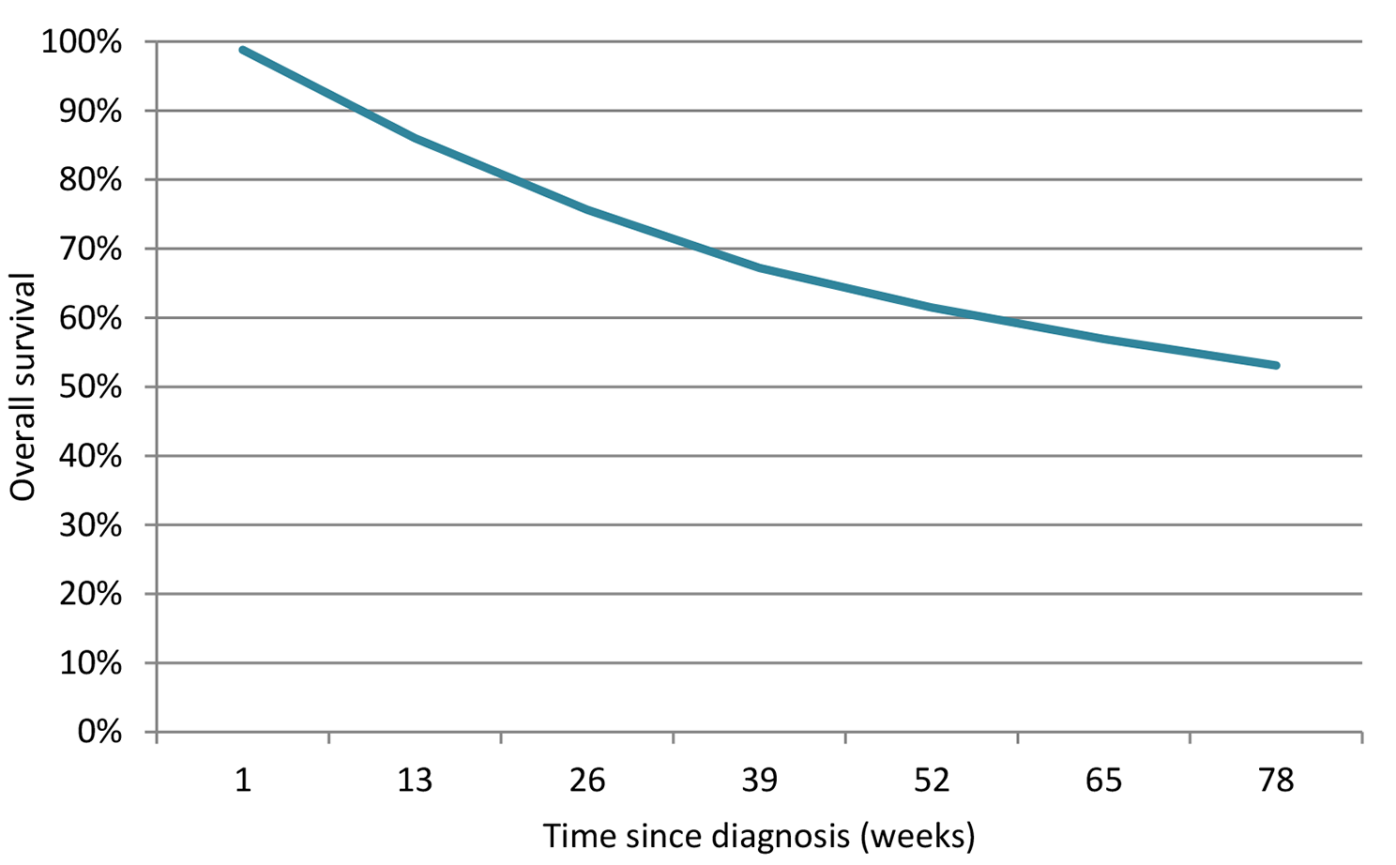
Individual prediction example of the final model. Survival function of a patient A with an incurable tumor, no previous HNSCC, cT1N0M1, 0 kg weight loss and WHO performance status 0

**Fig. 2:**
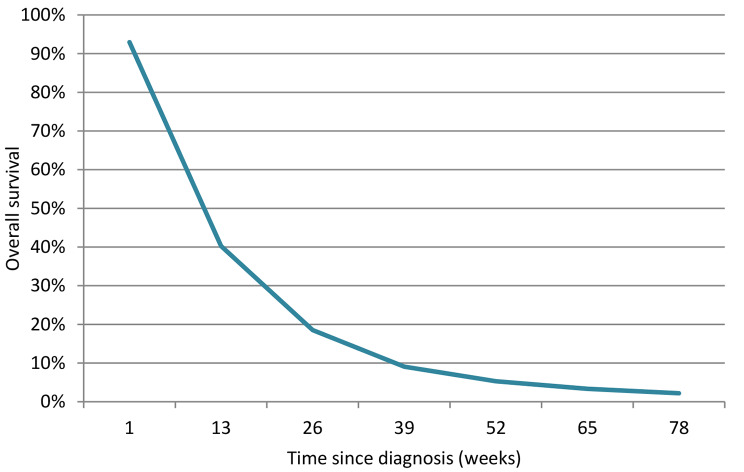
Individual prediction example of the final model. Survival function of a patient B with an incurable tumor, two previous HNSCC, cT4N0M1, 10 kg weight loss and WHO performance status 2

The prognostic variables: the reason for entering the palliative phase (incurable tumor / refusal), the number of previous HNSCC, cT, cN, cM, weight loss in the 6 months before diagnosis, and the WHO performance status were significant predictors of OS in the multivariable analysis and were included in the final model. The multivariable HRs can be found in Table 1, the coefficients of the full model equation can be found in Table 2. The internally validated C-statistic was 0.66 indicating moderate discriminative ability. The model showed some optimism, with a shrinkage factor of 0.89. Individual prediction examples are shown in Figs. 1 and 2.

**Table 2:** Coefficients of the full model equation

Characteristic	Coefficients (β)	Shrinked coefficients (β)
**No. previous HNSCC (0)**	-	
1	0.5641	0.5027918
2	0.5471	0.4876394
3 / 4	0.6946	0.6191086
**Reason of palliative phase** **(incurable tumor)**	-	
refusal of curative treatment	0.3285	0.2927975
**cT (0)**	-	
1	-0.2606	-0.2322771
2	-0.2243	-0.1999223
3	0.0984	0.08770556
4	0.3418	0.304652
**cN (0)**	-	
1	0.1605	0.1430563
2	0.2000	0.1782633
3	0.5154	0.4593846
**cM (0)**	-	
1	0.4154	0.370253
**Weight loss in the in the 6 months before diagnosis kg**	0.0279	0.02486774
**WHO performance (0)**	-	
1	0.4343	0.3870988
2	0.5884	0.5244507
3 / 4	1.4251	1.270215

## Discussion

This study aimed to develop a prognostic model for HNSCC patients in the palliative phase that predicts the individual survival based on prognostic factors which are available in most clinics. The internally validated C-statistic was 0.66, suggesting a moderate discriminative performance [24, 26].

### Predictors of survival

Patients who refused curative treatment had a better survival probability in the univariable analyses (non-significant), while in the multivariable analyses refusing curative treatment was significantly harmful. This could be explained by the fact that patients who refused curative treatment had for example significant less previous HNSCC, and significant lower ACE-scores than the patients with an incurable head and neck tumor. In the multivariable analyses this effect is corrected for all other variables resulting in significant worse survival probability (HR 1.39, 95% CI: 1.06; 1.82). The presence of previous HNSCC was related to a significant worse prognosis in both the univariable and multivariable analysis, which is in accordance with previous studies [27, 28]. Although nine candidate predictors showed an univariable significant effect on mortality, seven predictors were included in the final multivariable model. For example, a higher BMI was significantly related to a better prognosis in the univariable analyses, which is in agreement with earlier research [29, 30]. However, this variable was excluded in the multivariable analysis. A possible explanation for this is the correlation with the variable weight loss in the 6 months before diagnosis, which was significantly harmful in both univariable and multivariable analysis. As a result of this the effect of BMI is already implicitly included in the effect of weight loss. While earlier research among curative patients reported a significant harmful effect of tobacco and alcohol consumption on survival [18, 31, 32], these variables had no significant effect on survival in the current study. This could be due to the short life-span of the patients included in this study with a median OS time of 15 weeks: they simply do not live long enough to experience a harmful effect of smoking or alcohol consumption in the last phase of life. Similarly, Argiris et al. observed no significant effect on the 2-years survival related to tobacco and alcohol consumption in patients with metastatic and recurrent HNSCC [33]. Palliative treatment was not added as a possible prognostic factor due to confounding by indication. Estimating treatment effectiveness should be done in randomized controlled trial data and not in observational data to prevent this kind of confounding [24, 34, 35]. Although palliative treatment could not be added as a prognostic factor, an average treatment effect is assumed when estimating survival chances.

### Strengths and limitations

An important strength of this study was the consecutive design and the relatively large number of included patients (n = 656), the number of available variables (n = 20) and the high number of events (n = 647). In addition, the data is collected according to a comprehensive validation protocol developed to guarantee the quality of the data. Linking our database with the MPRD minimalized lost to follow-up which resulted in only three missing cases. The amount of missing data was limited except for serum hemoglobin (62.3% missing). To avoid complete case analysis resulting in data loss, multiple imputation was used to handle missing values except for hemoglobin [36–38]. Although 20 variables were added, there were also variables missing like human papillomavirus and Epstein-Barr virus status. Also, there was a relative low palliative treatment rate (29.3%) which should be taken into consideration during external validation.

### Clinical implications and future perspectives

Prior to clinical implementation, the model should be externally validated and subsequently integrated in an online tool that meets patients’ and doctors’ user needs. These needs should be thoroughly examined beforehand. An effective way of doing this is by qualitative research which aims to gain a deep understanding of a specific topic. We recently published our focus group study on life-expectancy and the prognostic model OncologIQ among curatively treated HNSCC patients [10]. OncologIQ, predicts the 1- to 10 year OS probability in patients who are eligible for curative treatment of HNSCC and was recently updated using the recommendations of patients included in our qualitative research [10, 18]. Next, an online tool was developed [39] and its clinical impact is currently being evaluated in a clinical trial. It would be tempting to follow the same steps for the palliative prognostic model. However, the palliative model holds different and less prognostic factors than OncologIQ. It also seems likely that patients in the palliative phase have different information needs when it comes to sharing prognostic information [10]. Therefore this should be further explored using qualitative research. Simultaneously, doctors should be involved in this process which will facilitate clinical implementation. The models’ estimation remains a probability calculation and the actual outcome could be better or worse. It also remains unclear to what extend patients and even healthcare professionals understand the uncertainty around these estimates and what would be the best way to communicate this uncertainty [40, 41]. This should also be explored beforehand. Based on these results an implementation plan will be developed. Predicting the individual palliative prognosis more accurately facilitates more realistic prognostic discussion. This could enable patients and their caregivers to prepare themselves for the approaching end of life. Furthermore, palliative care planning, including the decision whether to start palliative treatment, could be optimized.

## Conclusions

This study enabled the development and internal validation of a prognostic model that predicts OS probability in HNSCC patients in the palliative phase. This model facilitates personalized prognostic counseling in the palliative phase. External validation and qualitative research is necessary before widespread use in patient counseling and end-of-life care.

## Data Availability

The datasets generated and/or analyzed during the current study are not publicly available. The full dataset could contain information that might compromise research participants’ privacy and/or their conditions of consent. The data that support the findings of this study may be available on reasonable request from the corresponding author [AH].

## Abbreviations

ACE-27: Adult Comorbidity Evaluation-27
AJCC: American Joint Committee on Cancer
BMI: Body Mass Index
CI: Confidence Interval
HNSCC: Head and Neck Squamous Cell Cancer
HR: Hazard Ratio
MPRD: Municipal Personal Records Database
NCR: Netherlands Cancer Registry
RONCDOC: Rotterdam Oncological Documentation
SPSS: Statistical Package for the Social Sciences
TRIPOD: Transparent Reporting of a multivariable prediction model for Individual Prognosis and Diagnosis
